# Breaking the Barrier: The Role of Gut Epithelial Permeability in the Pathogenesis of Hypertension

**DOI:** 10.1007/s11906-024-01307-2

**Published:** 2024-04-25

**Authors:** Matthew Snelson, Tim Vanuytsel, Francine Z. Marques

**Affiliations:** 1 Hypertension Research Laboratory, School of Biological Sciences, Monash University, Melbourne, Australia; 2 Victorian Heart Institute, Monash University, Melbourne, Australia; 3 Translational Research Center for Gastrointestinal Disorders, Department of Chronic Diseases and Metabolism, KU Leuven, Leuven, Belgium; 4 Department of Gastroenterology and Hepatology, University Hospitals Leuven, Leuven, Belgium; 5 Heart Failure Research Group, Baker Heart and Diabetes Institute, Melbourne, Australia

**Keywords:** Intestinal permeability, Colonic permeability, Hypertension, Tight junction proteins, Gut, Microbiome

## Abstract

**Purpose of the Review:**

To review what intestinal permeability is and how it is measured, and to summarise the current evidence linking altered intestinal permeability with the development of hypertension.

**Recent Findings:**

Increased gastrointestinal permeability, directly measured *in vivo*, has been demonstrated in experimental and genetic animal models of hypertension. This is consistent with the passage of microbial substances to the systemic circulation and the activation of inflammatory pathways. Evidence for increased gut permeability in human hypertension has been reliant of a handful of blood biomarkers, with no studies directly measuring gut permeability in hypertensive cohorts. There is emerging literature that some of these putative biomarkers may not accurately reflect permeability of the gastrointestinal tract.

**Summary:**

Data from animal models of hypertension support they have increased gut permeability; however, there is a dearth of conclusive evidence in humans. Future studies are needed that directly measure intestinal permeability in people with hypertension.

## Introduction

In the last decade, it has emerged that intestinal permeability may be a risk factor for cardiometabolic diseases, contributing to inflammatory sequalae and subsequent disease progression [1]. The intestinal wall is lined by a monolayer of epithelial cells that have a crucial role in separating the gut microbiome from the host. When the gut epithelial barrier is disrupted, lipopolysaccharide (LPS) and other detrimental microbial products enter the host's circulation, leading to the activation of systemic inflammation [2]. Higher inflammatory state is believed to be involved in the development and maintenance of hypertension [3], but many of the mechanisms that start and then maintain this elevated inflammatory state are yet to be determined. Increased permeability of the gut barrier has been observed to occur with a variety of physiological stressors including heat stress [4, 5], exercise stress [6], psychological stress [7], excessive dietary fat intake [8], lack of fibre [9], and sleep deprivation [10] – many of these are also risk factors for hypertension [11, 12]. Here, we review what intestinal permeability is and how it is measured, and the current evidence that links it to the development of experimental and clinical hypertension.

## The Gut Epithelium Barrier

The human gastrointestinal tract has a surface area of around 32m^2^ [13], equivalent to a typical studio apartment. This surface is lined with a single layer of epithelial cells, kept in close proximity by tight junction proteins, adherens junction proteins, and desmosomes [14]. Moreover, goblet cells, specialised cells intercalated with epithelial cells, synthesise and secret the mucin glycoprotein MUC2 as the outermost layer of the intestinal barrier in contact with the gut microbiota [15]. Mucins are the major component of mucus and form a protective layer to the epithelial cells [15]. They serve as a localised niche for some commensal gut microbiota that specialise in binding to and degrading mucin glycans [15]. In healthy conditions, there is a tight balance between the production of and degradation of mucins; however, in unhealthy conditions (e.g., absence of dietary fibres), some of these bacteria can have a high turnover of glycan degradation, degrading the mucus layer and contributing to the breakdown of the epithelial layer in the process [9]. Together, the epithelial cells, intercellular proteins, and mucins form a layer that plays important physical and functional roles in separating the luminal microbiome from the immune cells that inhabit the lamina propria [16]. The intestinal epithelium barrier provides a defence against the entry of harmful substances, such as pathogens and toxins, whilst simultaneously permitting sufficient absorption of nutrients, electrolytes, and water from the gastrointestinal lumen [14]. While the full impacts of a disrupted barrier are still being elucidated, when it is disrupted, immune response pathways are activated in intestinal tissue [9]. Moreover, this could lead to the passage of microbial molecules, such as the bacterial surface LPS, from the intestinal lumen to the systemic circulation, where it could trigger a series of inflammatory responses that contribute to the low-grade chronic inflammation observed in hypertension. While measurement of LPS in healthy individuals is difficult due to its low levels and issues with contaminated lab equipment, inhibition of LPS-receptor, the toll-like receptor 4 (TLR4), reduces blood pressure (BP) and inflammatory markers (e.g., IL6, macrophage infiltration to the kidneys) in experimental models of hypertension [17, 18]. It is also possible to hypothesise that an impaired gut epithelial barrier will impact the regulation of sodium and water absorption, which could also have an effect on BP.

### Permeability of the Gut Barrier

**Fig. 1:**
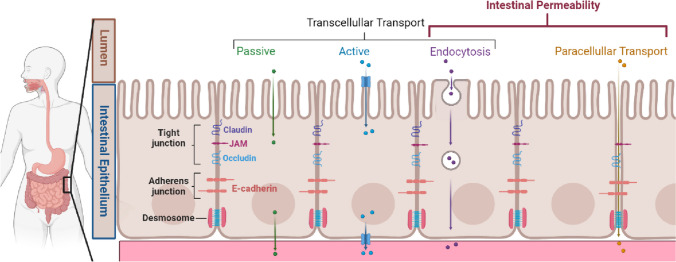
Overview of intestinal transport mechanisms and epithelial barrier permeability

Luminal contents can cross the gastrointestinal epithelium via either transcellular or paracellular mechanisms, depending on molecular size, charge, and hydrophobicity. Molecules may cross transcellularly in a number of mechanisms: 1) passive transport, as is the case for small compounds that may diffuse across the epithelial cell membrane; 2) active transport utilising substrate specific cell surface receptors (e.g., as is the case for monosaccharides and amino acids); and 3) endocytosis, whereby larger peptides and proteins are absorbed via vesicles (Fig. 1). Transcellular endocytosis also appears to be a mechanism by which bacterial components, such as LPS, bacterial extracellular vesicles or even whole bacteria, may cross the gastrointestinal barrier [19••, 20]. In addition to the transcellullar mechanisms described here, molecules may pass via the paracellular route, discussed in further detail below (Fig. 1). The paracellular and the transcellular endocytosis pathways are the most relevant for intestinal permeability, with most research to date focused on the paracellular pathway [21].

Tight junction proteins, adherens junction proteins, and desmosomes facilitate close intercellular contact among the epithelial cells of the intestinal tract. Luminal contents can pass across the epithelial barrier via transcellular passive, active or endocytosis mechanisms, or via paracellular pathway. Intestinal permeability refers to movement of molecules via the transcellular endocytosis or paracellular pathways.

### The Paracellular Pathway

Cell-to-cell contact between the epithelial cells of the gastrointestinal tract is maintained by tight junction proteins (e.g., occludin, zonula occludens-1, and claudin-2), adherens junction proteins (e.g., E-cadherin), and desmosomes. Paracellular permeability is regulated by the tight junction proteins, which determine the size and charge of molecules that are able to pass between the epithelial cells [22]. The intracellular peripheral membrane proteins include the zonula occludens (ZO) family, ZO-1 (also known as tight junction protein-1 [TJP1]), ZO-2, and ZO-3 as well as cingulin, whilst the transmembrane proteins include occludin, tricellulin, and members of the claudin family. There are a total of 27 claudins in mammals, although not all claudins have relevance for the intestinal barrier [19••]. These tight junction proteins permit the paracellular movement of molecules via two different pathways, which have been named the pore or leak pathway.

The pore pathway is defined by claudin proteins which form a charge and size specific channel in the tight junction [23], permitting molecules up to < 8 Å in size to pass between epithelial cells [24]. Thus, a sodium ion, which is positively charged with a size ~ 1 Å, may pass freely through the pore pathway, whilst a glucose molecule (size 9 Å) would be unable. Claudin-2 (CLDN2) and claudin-15 (CLDN15) are the main pore forming claudins expressed in the gastrointestinal tract and both of these claudins form cation specific pores [25, 26], permitting the paracellular transport of sodium ions. The cytokines IL-1β, IL-6, IL-13 and IL-22 have been demonstrated to alter intestinal transcription of CLDN2 [27–30], although the impact this has on subsequent pore pathway permeability has not been fully elucidated. A possibility is that sodium activation of the immune system and associated produced of these cytokines leads to higher expression of CLDN2, which is associated with higher inflammation in an experimental model of colitis [31]. Moreover, lack of CLDN15 in mice decreased sodium permeability [32]. Considering the intestine is where most of dietary sodium is absorbed, it is plausible to hypothesise that blocking intestinal CLDN2 and CLDN15 may lead to lower sodium uptake and, thus, BP. While no variants in/near these genes have been associated with hypertension in genome-wide association studies, it is possible that these are associated with salt-sensitive hypertension.

The leak pathway is considerably larger, allowing molecules up to 100 Å in size through, with no restrictions on molecular charge [24]. Whilst the specific molecular structure of this pathway is less understood than the pore pathway, it is thought to be regulated by occludin, tricellulin, ZO-1, and perijunctional actomyosin [19••, 33], although there is evidence that alterations in tricellulin expression may affect permeability of this pathway [34]. Myosin light chain kinase splice variant 1 (MLCK1) has been shown to trigger endocytosis of occludin, leading to increased permeability. The expression and activity of MLCK1 can be increased by a number of cytokines including TNF-α [35–37] and IL-1β [38], providing evidence for the role of the immune system signalling in affecting paracellular permeability.

In addition to the leak and pore pathways, there is also the unrestricted (or apoptotic) pathway, where severe damage to the gut mucosa results in death of epithelial cells and permits unrestricted paracellular transport of large molecules and bacteria. This epithelial damage and subsequent unrestricted paracellular permeability has been observed in graft vs host disease and gastrointestinal conditions such as Crohn's disease and ulcerative colitis [39], and may play a role after acute cardiovascular events, such as after a stroke, where damage to intestinal epithelial cells is severe [40].

## Measuring Intestinal Permeability

Direct measurement of intestinal permeability involves the assessment of a molecule or group of molecules that moves from one side of the intestinal epithelium to the other side. Conversely, indirect measurements investigate biomarkers that are present in collected samples, most commonly blood, that may be utilised for assessment of intestinal permeability.

### *Ex vivo *Measurement

The Ussing chamber technique is a sophisticated method which involves isolating a segment of the gut and mounting it between two halves of a chamber, with each half filled with a physiological solution. The chamber is designed to separate the luminal side from the basolateral side of the epithelium and, to assess permeability, molecules are placed in the luminal chamber, and the flux of these markers to the basolateral side is measured as an indicator of permeability of the epithelium [41]. The size of the molecules selected may permit understanding of the different routes of transport, with molecules such as inulin, polyethylene glycols (PEGs) and Fluorescein Isothiocyanate-Dextran (FITC-Dextran) being utilised for assessment of the paracellular route, whilst larger molecules such as horseradish peroxidase can be utilised for assessment of the transcellular route [21]. The Ussing chamber technique is often utilised in animal studies where intestinal tissues may be obtained following sacrifice of the animals [41, 42] and in human studies of intestinal diseases where endoscopic biopsy samples may be obtained [43]. However, the procurement of endoscopic biopsy samples may prove difficult outside the context of intestinal diseases where endoscopy may be indicated for assessment of disease progression.

### *In vivo* Measurements

Assessment of paracellular permeability is conducted *in vivo* by assessment of the plasma or urine of orally digested probes. There are many different substances which may be utilised depending on the experimental question being posed, including FITC-Dextran, PEGs, Chromium-51-labeled ethylenediaminetetraacetic acid (^51^CrEDTA), lactulose, mannitol, sucralose and sucrose [21]. The key features for probes is that they must be an adequate size to assess the route of permeability being assessed and freely filtered into urine where they can be measured. The FITC-Dextran permeability test, first described by Tagesson et al. in 1978 [44], is commonly utilized in murine studies due to its relative ease. In this assay, animals are orally gavaged with a solution containing FITC-Dextran (4-kDa), blood is collected and fluorescence measured using a typical laboratory plate reader with fluorescence detection [45]. If a timepoint for blood collection of 1 h post ingestion is selected, the FITC-Dextran assay will represent small intestinal permeability, whilst longer timepoints (4–6 h) will represent whole gut permeability. Animals are dosed according to body weight (usually at 400–600 mg/kg body weight [46–48]), though an argument has recently been made for dosing to be conducted according to lean body mass, at least in obese animal models [49]. Whilst this technique can be applied to humans, in practice it is not used, likely related to the costs associated with the dosages required. PEG and ^51^CrEDTA are both resistant to microbial fermentation in the colon, and, thus, can be utilised for assessment of whole gut permeability [50]. Measurement of intestinal permeability using ^51^CrEDTA has been utilised in rodent [51] and human studies [52–54], however does require the participant to be exposed to a small amount of radiation [55]. Recently, a new protocol using non-radioactive ^52^Cr-EDTA has been proposed [56].

PEGs span a range of molecular sizes and are assessed in urine by high-pressure liquid chromatography [57]. The assessment of intestinal permeability using PEGs has been shown on have a high agreement with the dual sugar (lactulose/rhamnose) test [58], which is the most popular methodology for assessment of intestinal permeability in humans.

## Dual and Multisugar Tests

The most popular *in vivo* direct measurement of intestinal permeability has been the dual sugar test. In this test, a bolus of monosaccharide (mannitol or L-rhamnose) and disaccharide (lactulose) is consumed orally. In the intestine, the smaller monosaccharides pass through the paracellular pore pathway which permits molecules < 8 Å in size, which the disaccharide cannot pass through [24]. If there is disruption of the tight junction proteins, then these larger molecules can pass through the leak pathway, and the ratio in urine or blood between lactulose and mannitol or L-rhamnose, is used to represent permeability. Urine collected up to 2 h post ingestion is considered to represent exclusively small intestinal permeability [57, 59], although some studies use up to 5–6 h post ingestion to assess small intestinal permeability [60, 61•]. Recently it was suggested that serial plasma measurements (i.e. hourly) may provide greater sensitivity to transient intestinal permeability changes compared with urine collections [5]. Whilst mannitol has traditionally been utilised as the monosaccharide in the dual sugar test, mannitol is present in the regular diet and may, thus, contaminant the results [62]. ^13^C mannitol and L-rhamnose are alternative monosaccharides that avoid this dietary contamination issue [63]. While useful for measuring small intestinal permeability, lactulose, mannitol and L-rhamnose are all fermented by the gut microbiota when they reach the colon and are thus unreliable for the assessment of specifically colonic permeability.

The small intestine and large intestine (especially the colon) are now recognised to be vastly different anatomically, in terms of physiological functions, pH, and number of bacteria [64]. The permeability difference between these regions is under-appreciated, with the dual sugar tests only assessing small intestinal permeability. A multisugar test has been developed to assess both small intestine and colonic permeability [65, 66]. In this case, monosaccharides (L-rhamnose and erythritol) and disaccharides (lactulose and sucralose) are consumed orally. Permeability in the small intestine is assessed with L-rhamnose and lactulose as per the dual sugar test, whilst in the colon this can be assessed with the non-fermented erythritol and sucralose. Commonly a time period of up to 5 h post-ingestion is considered to represent the small intestine, whilst between 5 to 24 h represents the colon [60, 67]. However, some studies have utilised between 8 and 24 h to represent the colon [68, 69], because there is a large intra-individual variation in small intestinal transit time (from 50–460 min) [70]. Given this wide variation, for studies that want to exclusively assess small intestinal, and not colonic, permeability, it may be prudent to focus on the urine collection of up to 2 h post-ingestion.

### Blood Biomarkers of Intestinal Permeability

Whilst the above methods represent direct measurement of molecules that are passing from the luminal side of the gastrointestinal epithelium to the basolateral side for measurement in blood or urine, there is also interest in finding suitable biomarkers for intestinal permeability and many have been proposed. The presence in the blood of LPS, from Gram negative bacteria, has long been considered a clear indicator of intestinal barrier dysfunction [71]. However, there are mounting concerns that the assay used for LPS is not accurate at measuring LPS at lower levels seen in non-septicaemia [72]. Liposaccharide binding protein (LBP) is an endogenous protein produced by the body in response to the presence of LPS, and has been suggested as a more reliable marker [73]. Indeed, a recent study in an obese cohort assessed the correlation between the dual sugar test with six biomarkers (faecal albumin, calprotectin, and zonulin, and plasma intestinal fatty acid binding protein [I-FAPB], LBP and zonulin) [61•]. LBP was the only plasma marker consistently correlated with *in vivo* permeability measurement in both participants with healthy and higher body weight, showing a variation according to the group studied, from r = 0.423 in those with a healthy body weight up to r = 0.813 in the cohort with obesity [61•]. This shows that LBP may not be a reliable marker for all healthy and disease states.

Disconcertedly, this study found no correlation with two other commonly used gut permeability plasma biomarkers, I-FAPB or zonulin [61•]. I-FAPB is a protein present in differentiated enterocytes, and damage to the epithelial layer results in this protein being released from cells – hence, it is considered an indicator of epithelial damage [74]. Increased I-FAPB has been observed in severe intestinal conditions such as necrotizing enterocolitis [75] and intestinal ischemia [76]. With regards to zonulin, there is mounting evidence that commercially available zonulin ELISA kits are not actually measuring zonulin, but rather other related proteins with unknown function [77, 78]. A recent paper compared zonulin levels with colonic paracellular permeability using Ussing chambers in IBS patients and found no correlation [79]. It has been recommended that studies that assess gut permeability using zonulin measured by these ELISA assays be interpreted with caution.

D-lactate is a byproduct of bacterial carbohydrate fermentation and is minimally produced by human metabolism, with serum levels representing translocation from the gut lumen and thus gut permeability. Serum D-Lactate levels are elevated in Crohn’s disease [80], critically ill patients with gastrointestinal failure [81]. Serum and urinary levels of D-lactate are elevated in diabetes [82], suggesting this marker may be appropriate outside the context of gastrointestinal conditions. Not all bacteria produce D-lactate, with Lactobacilli being the primary D-lactate producers in the human gastrointestinal tract [83]. There is a dearth of evidence as to whether the composition of the gut microbiota and relative abundance of D-Lactate producers affects the reliability of D-Lactate as a marker of gut permeability. Diamine oxidase (DAO) is an enzyme involved in histamine metabolism that is produced by the intestinal mucosa, as well as the kidneys and placenta [84]. Serum DAO levels have been proposed to be a marker of intestinal barrier integrity [85] and is elevated in Crohn’s disease [80]. However, DAO levels are affected by factors such as diet [86], alcohol consumption [87], sex [88], menstrual cycle [89] and pregnancy status [90] which challenge it’s reliability as a biomarker [91]. In summary, more sensitive and accurate markers for intestinal permeability are needed.

## Gut Permeability and Hypertension

There is some evidence for alterations in intestinal permeability in hypertension that has been demonstrated in experimental models of hypertension, as well as studies comparing hypertensive and normotensive individuals. As discussed in the previous section, there are different methodologies available for the assessment of intestinal permeability, each with their own benefits and flaws. Overall, the evidence to date, particularly from animal studies (summarised in Table 1), supports the presence of intestinal barrier dysfunction in hypertension. Whether increased gut permeability per se has a causative role in the pathogenesis of hypertension or is a consequence of the disease process remains to be elucidated.

**Table 1:** Summary of Gut Permeability Measures in Animal Models of Hypertension

**Study Ref**	**Animal Model of Hypertension**	**Comparison Animal Model**	**Measure of gut permeability**	**Other relevant measurements**
*SHR rat:*				
Santisteban et al. 2017 [92]	Pre-hypertensive 4-week-old SHRs	4-week-old WKY rats	ND: FITC-Dextran *Protein expression (WB)* *SI:* ↓ ZO-1, claudin-4, cingulin. ND: occludin *Colon:* ↓ occludin, ZO-1, claudin-4, cingulin	
Santisteban et al. 2017 [92]	20-week-old SHRs	20-week-old WKY rats	↑ FITC-Dextran *Protein expression (WB)* *SI:* ↓ occludin, ZO-1, claudin-4. ND: cingulin *Colon:*↓ occludin, ZO-1, cingulin. ND: Claudin-4	↓ goblet cells in small intestine ↑ Fibrotic area in small intestine ↓ intestinal blood perfusion
Jaworska et al. 2017 [93]	24–26-week-old SHRs	24–26 week old WKY rats	↑ portal vein TMA following intracolonic TMA infusion *Protein expression (IF):* ND: colonic occludin or ZO-1	↓ intestinal blood flow
Wang et al. 2021 [94]	Pre-hypertensive 5-week-old SHRs	5-week-old WKY rats	*Colon Ussing Chamber* ↑ paracellular permeability, ND: transcellular permeability *Protein expression (WB)* *Colon:* ↓ claudin5, occludin, ZO-1	
*Ang II infusion model:*				
Santisteban et al. 2017 [92]	8-week-old Sprague Dawley Rats infused with Ang II	8-week-old Sprague Dawley Rats infused with saline	↑ FITC-Dextran *Protein expression (WB)* *SI:* ↓ occludin, ZO-1, cingulin. ND: claudin-4 *Colon:* ↓ occludin, ZO-1, cingulin, claudin-4	
Kim et al. 2018 [48]	C57BL/6 mice infused with Ang II (4-weeks)	C57BL/6 mouse infused with saline	↑ FITC-Dextran G*ene expression*:↓ occludin, ZO-1. ND: claudin-4	
Kaye et al. 2020 [96]	C57BL/6 J mice infused with Ang II (4-weeks)	C57BL/6 J mouse infused with saline	G*ene expression:* ↓ ZO-1	↑ TNF-α and IL-6 intestinal gene expression
*DOCA salt model:*				
Robles-Vera 2020 [97]	Male Wistar rats DOCA-salt induced hypertension	Male Wistar rats	↑ plasma LPS G*ene expression*: ↓ occludin and ZO-1	G*ene expression*: ↓ mucin-2 and mucin-3

**Legend**: Ang II = Angiotensin II, IF = Immunofluorescence, IL-6 = Interleukin 6, LPS = Lipopolysaccharide, SHR = Spontaneously Hypertensive Rats, TMA = Trimethylamine, TNF-α = Tumour Necrosis Factor Alpha, WB = Western Blot, WKY = Wistar-Kyoto (rats), ZO-1 = Zonula occludens-1. ND = No Difference, ↑ = Increased ↓ = decreased (in hypertension relative to control)

### Evidence from Experimental Animal Models of Hypertension

The first published study to investigate gut permeability in animal models of hypertension was Santisteban et al*.* (2017), who observed increased gut permeability (assessed by the FITC-Dextran assay) in 20 week old, but not 4 week old, spontaneously hypertensive rats (SHR) relative to age-matched Wistar Kyoto (WKY) rats [92]. This suggests that hypertension may be established prior to the increase in intestinal permeability; however, further experiments to pinpoint when this happens exactly are needed to confirm this hypothesis. Shortly after, Jaworska et al*.* (2017) observed increased gut permeability in a unique assay which assessed the rise in portal vein trimethylamine (TMA, a metabolite produced by the gut microbiota) concentrations following intracolonic TMA infusion in 24–26-week old SHRs relative to age-matched WKY rats [93]. Both studies observed these findings were ameliorated with angiotensin converting enzyme (ACE) inhibition. A more recent study in 5-week-old SHRs utilised Ussing chambers for assessing colonic permeability [94]. They noted an increase in paracellular, but not transcellular, permeability [94], which supports alterations in tight junction proteins. Indeed, the levels of several tight junction proteins, measured by Western blots, including occludin and ZO-1 were decreased in hypertensive models in some studies [92, 94], but not others, where differences in colonic occludin or ZO-1 between hypertensive and normotensive animals was not detected when these were assessed by immunofluorescence [93]. Moreover, several studies in the SHR model observed decreased intestinal blood flow [92, 93] and increased intestinal fibrosis [92, 95].

Several studies have also utilised angiotensin II infusion to induce hypertension to assess the effects of hypertension on gut permeability. Rodents infused with angiotensin II had greater gut permeability as assessed by FITC-Dextran [92, 48] and decreased intestinal gene expression of tight junction proteins including ZO-1, particularly on a low fibre diet [48, 96], which was accompanied by increased intestinal expression of the inflammatory cytokines TNF-α and IL-6 [96]. Similarly, a study that utilised the DOCA-salt model of hypertension observed increased plasma LPS and decreased colonic gene expression of occludin and ZO-1 [97]. Thus, evidence from experimental animal models has generally supported the presence of increased paracellular permeability in hypertensive models.

### Evidence from Human and Non-Human Primates

Kim et al. [48] examined biomarkers of intestinal permeability in a high BP group with a reference group (n = 35) (Table 2). Participants were grouped according to office SBP, irrespective of antihypertensive medication; as such only one-third of the reference group were normotensive, with the remainder having treated hypertension. Similarly, the high BP group contained participants with untreated hypertension, poorly-controlled hypertension, and resistant hypertension. This study found the high BP group had increased plasma levels of I-FAPB, zonulin, and LPS [48]. Similarly, a cross-sectional study in young adults (18–25 years old, n = 96) found those with hypertension had higher serum zonulin levels than normotensive participants [98]. Li et al. conducted a retrospective analysis of 357 gastroenterology inpatient records who had had measurements of serum DAO, LPS, and D-lactate and compared hypertensive to normotensive inpatients, regardless of antihypertensive medication status [99]. This study used cutoffs of ≥ 15, ≥ 20, and ≥ 10 U/L to define elevated levels of DAO, LPS, and D-lactate, respectively, and reported that a greater percentage of hypertensive patients met the criteria for elevated LPS and DAO, but not D-lactate, compared to normotensive patients.

**Table 2:** Summary of Gut Permeability Measures in Humans and Non-Human Primates with Hypertension

**Study Ref**	**Study Type**	**Hypertensive Cohort**	**Control Cohort**	**Measure of gut permeability**
Kim et al. 2018 [48]	Cross-sectional	SBP ≥ 140 mm Hg irrespective of participant’s antihypertensive drug regimen (n = 18)	SBP ≤ 130 mm Hg irrespective of participant’s antihypertensive drug regimen (n = 17)	↑ plasma I-FAPB ↑ plasma LPS ↑ plasma Zonulin
Ntlahla et al. 2021 [98]	Cross-sectional	SBP ≥ 140 mmHg and/or DBP ≥ 90 mmHg (n = 27)	SBP ≤ 120 mm Hg and DBP ≤ 80 mm Hg (n = 69)	↑ plasma Zonulin
Li et al. 2021 [99]	Retrospective Inpatient Records	SBP ≥ 140 mm Hg and/or DBP ≥ 90 mm Hg irrespective of participant’s antihypertensive drug regimen (n = 106)	SBP ≤ 130 mm Hg and DPB ≤ 80 mm Hg irrespective of participant’s antihypertensive drug regimen (n = 251)	↑ % with elevated DAO, ↑ % with elevated LPS, ND D-lactate
*Pregnancy:*				
Tomsett et al. 2020 [100]	Prospective Case–Control	Women who developed hypertension in late pregnancy (n = 19)	Women who remain normotensive during pregnancy (n = 36)	↑ plasma Zonulin at 28 weeks gestation ND plasma Zonulin at 16 weeks gestation
Wang et al. 2006 [101]	Cross-sectional	Pregnant women with pre-eclampsia (n = 13)	Women who remain normotensive during pregnancy (n = 13)	↑ plasma LPB at parturition
Mutluoglu et al. 2023 [102]	Cross-sectional	Pregnant women with pre-eclampsia (n = 22)	Pregnant women without pre-eclampsia (n = 22)	↓ plasma LBP and ↓ plasma Zonulin between 22–40 weeks of gestation
*Non-Human Primates:*				
Vemuri et al. 2022 [103•]	Cross-sectional	Vervet monkeys (n = 153)		LBP positively correlated with SBP and DBP
Vemuri et al. 2022 [103•]	Longitudinal	Adult male rhesus monkeys with SBP ≥ 120 mm Hg and/or DBP ≥ 80 mm Hg (n = 8)	Adult male rhesus monkeys with SBP ≤ 120 mm Hg and/or DBP ≤ 80 mm Hg (n = 8)	↑ plasma LBP at baseline and month 6, 18 and 27

**Legend**: DAO = Diamine Oxidase, DBP = Diastolic Blood Pressure, I-FAPB = Intestinal Fatty Acid Binding Protein, LBP = Lipopolysaccharide Binding Protein, LPS = lipopolysaccharide, SBP = Systolic Blood Pressure. ND = No Difference, ↑ = Increased ↓ = decreased (in hypertension relative to control)

Tomsett et al. compared women who proceeded to develop hypertension during pregnancy, including pre-eclampsia and gestational hypertension, with those that remained normotensive throughout pregnancy (n = 55) [100]. There was no difference in serum zonulin levels at 16-weeks of gestation, though at 28-weeks of gestation those who would become hypertensive had higher levels compared with normotensive women [100]. Similarly, a small study comparing 13 women with pre-eclampsia with 13 age-matched normotensive pregnant women showed that the women with pre-eclampsia had elevated LBP levels during hospital admission for parturition [101]. Paradoxically, a recent cross-sectional case control study comparing women with pre-eclampsia to healthy pregnant women at an obstetric visit during the third trimester observed that plasma LBP and zonulin levels were decreased in the women with pre-eclampsia compared with healthy controls (n = 44) [102]. There is conflicting evidence regarding intestinal permeability changes during pregnancy and hypertension.

One other interesting and relevant study to this discussion was undertaken in non-human primates. Vemuri et al. conducted a cross-sectional study in 153 vervet monkeys, and observed that LBP levels were correlated with both systolic and diastolic BP readings [103•]. In a second study by this group, 16 adult rhesus monkeys were assessed for hypertensive status using cutoffs for systolic and diastolic BP of 120 mm Hg and 80 mm Hg, respectively, a protocol that is suitable for use in non-human primates [104]. Hypertensive rhesus monkeys had higher LBP levels than normotensive rhesus monkeys at baseline, with LBP levels progressively elevating in the hypertensive group over the course of the study [103•]. This study was the first longitudinal assessment of a marker of intestinal permeability in non-human primates or humans and provides the first evidence that intestinal permeability does increase over time in the context of hypertension.

### Intestinal Permeability and Hypertension: The Chicken and Egg Question

A key question that remains is if hypertension leads to increased internal permeability, or if intestinal permeability, caused by other factors such as low fibre intake, gut dysbiosis, medication or comorbidities such as obesity, activates inflammatory processes that contribute to the development of hypertension. In the latter option, as BP increases, it could then damage the intestinal epithelium and further exacerbate permeability, and thus, BP. If intestinal permeability is indeed present in hypertension and involved in its pathophysiological mechanisms, another important question is if BP and its associated end-organ damage could be lowered by a reduction in intestinal permeability. For example, studies in mice using gut microbial metabolites that restore intestinal barrier function and reduce inflammatory cytokines also resulted in lower BP [96]. Whether this also happens in hypertensive patients is yet to be determined. A key limitation in the field is that only blood and faecal samples are available, and, as discussed above, the current biomarkers for intestinal permeability are not reliable enough to answer this question.

## Conclusion

Evidence from animal models has clearly demonstrated that there is increased gut permeability, assessed by direct measurement, in experimental models of hypertension. Evidence within human hypertension is limited, with several indirect biomarkers that are purported to be indicators of gut permeability increased in participants with hypertension. However, the reliability of these biomarkers is questionable. Whether hypertension leads to increased gut permeability, or vice-versa, has not been clearly established. It is possible that there is a bidirectional link between the two rather than a causal one-way relationship. A greater understanding of the role of gut permeability in the pathogenesis of hypertension may benefit targeted treatments to prevent and delay the scourge of elevated BP.
